# Characteristics of Serum Endocan Levels in Infection

**DOI:** 10.1371/journal.pone.0123358

**Published:** 2015-04-20

**Authors:** Kazunori Seo, Takatoshi Kitazawa, Yusuke Yoshino, Ichiro Koga, Yasuo Ota

**Affiliations:** Department of Internal Medicine, Teikyo University, Tokyo, Japan

## Abstract

**Objectives:**

Endocan is a newly recognized biomarker of sepsis. However, there have been no studies of the trends in endocan levels during infection and their associations with other clinical factors. The aim of this study was to assess the time course of endocan levels and the associations of endocan with clinical factors during infection by comparison with other biomarkers.

**Methods:**

Serum samples and blood cultures were obtained from patients who were diagnosed with infection from June 2013 to March 2014. Serum endocan, C-reactive protein (CRP), and procalcitonin (PCT) levels during four periods during infection were measured (day 0, day 1-2, day 3-5, and day 6-10).

**Results:**

A total of 78 patients were enrolled in this study. The median endocan level decreased by only 23% during infection, whereas both serum CRP and PCT levels decreased by more than 80%. Endocan levels were correlated to neither CRP levels nor PCT levels in each period. Endocan levels at day 0 in patients with bacteremia were higher than those without bacteremia (1.09 ng/mL vs 0.82 ng/mL, P=0.002), but neither CRP levels nor PCT levels at day 0 were different between the two groups. Areas under the receiver operator characteristic (ROC) curves of endocan, CRP, and PCT at day 0 were 0.662, 0.343, and 0.563, respectively. Positive blood cultures tended to be related to high endocan levels, but not significantly (odds ratio: 4.24, 95% CI: 0.99-10.34, P=0.05).

**Conclusions:**

In bacteremic cases, serum endocan levels in bacteremia tended to be higher than in non-bacteremic cases. Although endocan level was not identified as a prognostic factor of bacteremia, further prospective study concerning the relationship between serum endocan level and bacteremia would be needed.

## Introduction

Bacteremia is potentially life-threatening and thus requires early diagnosis and prompt administration of antibiotics to reduce mortality related to multiple organ failure. During the course of bacteremia, the time at which adequate antibiotic treatment is started is a key factor affecting survival[1]. However, bacteremia is identified in only about 30% of patients with sepsis [2].

C-reactive protein (CRP) and procalcitonin (PCT) are currently used as clinical indicators of inflammation and infection including bacteremia, and several other biochemical markers have been investigated for their ability to detect sepsis in an early, reversible phase [3]. Nonetheless, identification of an ideal biomarker capable of making a clear distinction between sepsis and SIRS is imperative [4].

Endocan is a proteoglycan that can be detected in human blood and is expressed on the surface of endothelial cells of lungs and kidneys [5]. Endocan is a marker of angiogenesis previously studied in various types of cancer [6,7,8,9,10,11,12]. Synthesis and release of this molecule are promoted by proinflammatory cytokines such as tumor necrosis factor (TNF)-α and interleukin (IL)-1β [5,13,14]. A few studies have shown that endocan can be acknowledged as a good marker of endothelial dysfunction and multiorgan failure in sepsis, and it can be accepted as a good marker of survival prognosis in sepsis [15]. In clinical practice, however, the usefulness and characteristics of endocan in infection have not been investigated. Changes in serum endocan levels through infection have not been analyzed.

In this study, the time course of serum endocan levels during infection and the associations between serum endocan levels and clinical factors, including blood culture-positivity, were investigated.

## Methods

### Patients

This prospective study was conducted in the Department of Internal Medicine of Teikyo University Hospital between June 2013 and March 2014. The subjects were patients aged ≥20 years admitted due to infection. Infection was defined as (1) systemic manifestations of infection (e.g., fever, chills and/or hypotension) with positive results of microbiological or serological tests, or (2) focal manifestations of infection with positive microbiological or radiological tests. The onset of infection was defined as the time of admission. Patients with pre-existing malignancy were excluded to avoid measurement inconsistencies. This study was approved by the Teikyo University ethics committee (the approval number:13–032). Written informed consent was obtained from all participants in this study.

### Data collection

Biomarkers were measured during the four following periods: day 0, on the admission day; day 1–2, between 1 and 2 days after onset of infection; day 3–5, between 3 and 5 days after onset of infection; and day 6–10, between 6 and 10 days after onset of infection. Within one hour, samples were centrifuged at 3000 rpm for 10 minutes, and sera were kept at -20°C until assayed. To measure serum endocan and PCT concentrations, enzyme-linked immunosorbent assays were used (LUNGINNOV Systems, Lille, France; CUSABIO, Wuhan, P.R. China). CRP concentrations were measured by latex agglutination assays (LSI Medience Corporation, Tokyo, Japan). In order to collect demographic information and clinical information, medical records were reviewed.

### Statistical analysis

Relationships between categorical variables were analyzed using Pearson’s chi-square test. Fisher’s exact test was used when the expected count was less than 5. Continuous variables were compared using the Mann-Whitney U test because the data did not follow a normal distribution. Kendall’s rank correlation coefficients were determined in order to evaluate the possible relationships among biomarkers. Logistic regression analysis was carried out to estimate odds ratios (ORs) and 95% confidence intervals (CIs). All *p* values were two-sided, and *p* < 0.05 was considered significant. All analyses were performed using PASW software for Windows (Ver.17.0).

## Results

### Demographic data on patients with infection

A total of 78 patients were enrolled in this study. The patients’ demographic data are shown in Table 1. Of these 78 patients, 40 were male (51%) and 38 were female (49%). The median age of the patients was 79 years (range, 23–97 years). As for pre-existing conditions, diabetes was the most common comorbidity (26% of patients). Primary infection sites were identified in 70 cases. The most frequent site was the respiratory tract (45%), and the second most frequent site was the urinary tract (21%). In all patients, the Pitt Bacteremia score was ≤4. Pathogens were detected in 47 cases. Only one patient died of meningitis with *Streptococcus pneumoniae*. There were 52 patients who had sepsis and 26 patients who did not have sepsis according to the consensus sepsis definition.

**Table 1:** Patients’ clinical characteristics.

Factors	Total (n = 78)
Age (years, median, IQR)	79 (72–84)
Sex (male/female)	40/38
Comorbidities	
Diabetes	20 (26)
Chronic heart failure	16 (21)
Cerebrovascular disease	10 (13)
Chronic obstructive pulmonary disease	8 (10)
Chronic kidney disease	6 (8)
Primary infection site	
Respiratory tract	35 (45)
Urinary tract	16 (21)
Catheter-related bloodstream infection	6 (8)
Skin and soft tissue	4 (5)
Intra-abdominal	4 (5)
Endocarditis	3 (4)
Meningitis	2 (3)
Unidentified focus	8 (10)
Pathogen (s)	
Bacteria	43 (45)
Gram-positive	21 (27)
Gram-negative	22 (28)
Fungus	1 (1)
Polymicrobial	3 (4)
Unidentified pathogen	31 (40)
Positive blood culture	29 (37)
Pitt Bacteremia score >4	0 (0)

Numbers and percentages of each case are displayed. Abbreviations: IQR, interquartile range.

### Changes in biomarker levels during infection and correlations with each biomarker

**Fig 1:**
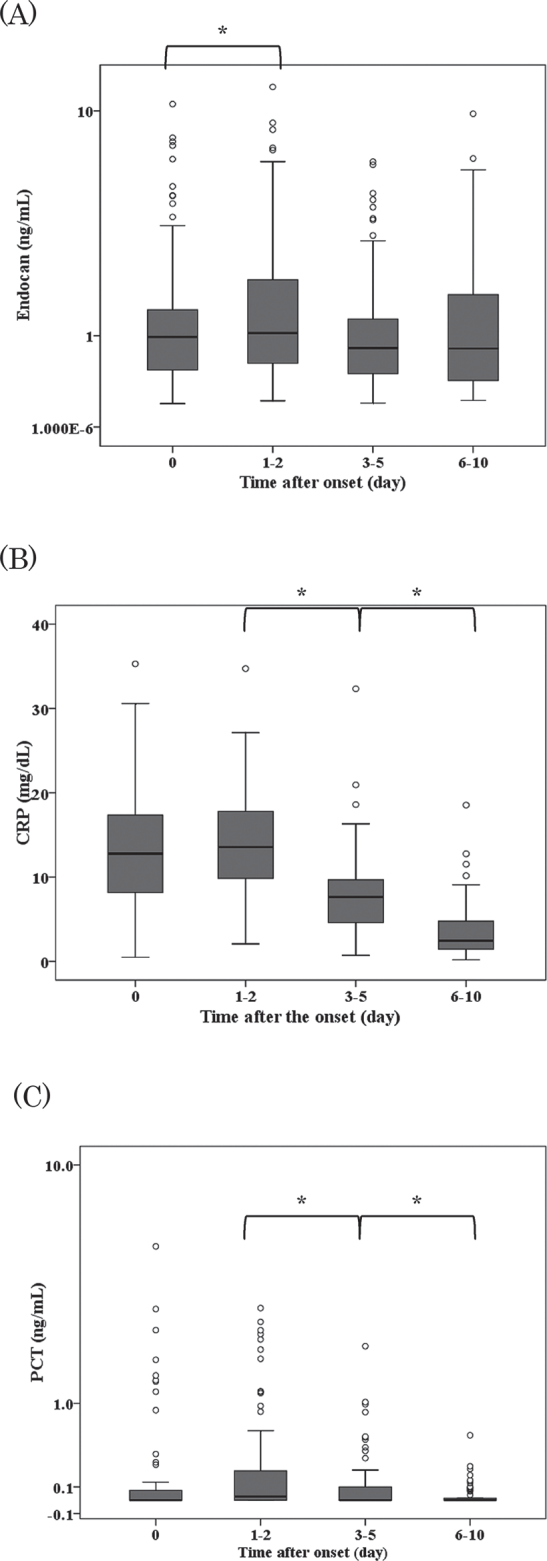
Trends of endocan (A), C-reactive protein (CRP)(B), procalcitonin (PCT)(C) levels in patients during infection. The boxes represent the interquartile range, and the transverse lines in the boxes represent the medians of each group. The vertical lines represent 1.5 of the interquartile range. The circles indicate outliers of each group. The y axes for endocan in (A) and for PCT in (C), but not for CRP in (B) are in log values.* P<0.001.

Changes in in biomarkers during infection were examined, and the serum levels of endocan, CRP, and PCT are shown in Fig 1. During the infection periods, a significant reduction of endocan levels was observed from day 1–2 to day 3–5 (median 1.03 ng/mL to 0.81 ng/mL, p<0.001). The reduction in the median endocan level during the whole infection period was 22.5%. On the other hand, significant reductions of both CRP and PCT levels were observed in two periods, from day 1–2 to day 3–5, and from day 3–5 to day 6–10. The reduction rates of the median CRP and PCT levels during the whole infection period were 82.0% and almost 100.0%, respectively.

**Fig 2:**
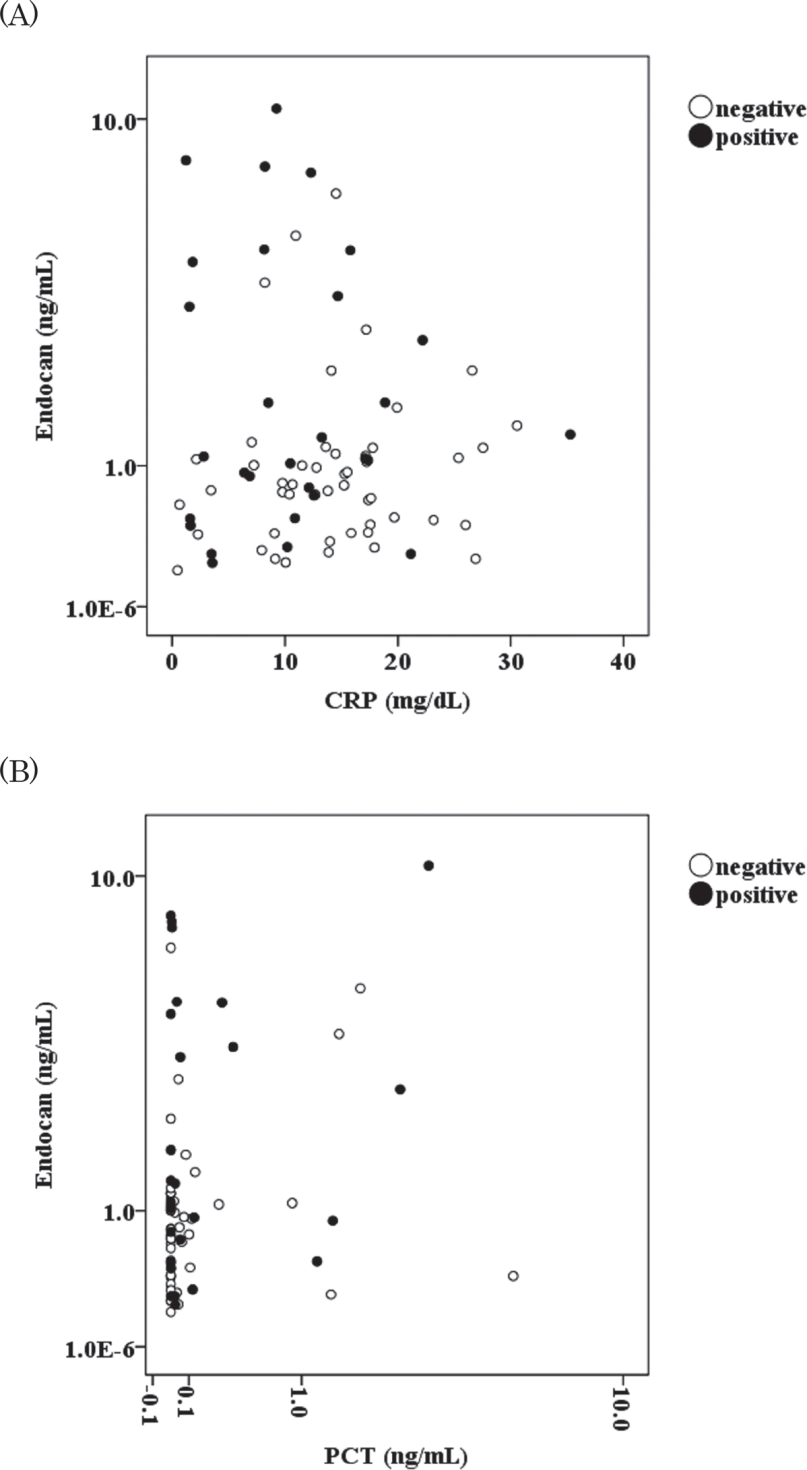
Correlations between the results for C-reactive protein (CRP) and endocan (A), and those for procalcitonin (PCT) and endocan (B). The black and white circles represent the levels of the markers in the blood culture-positive (n = 29) and-negative (n = 49) groups, respectively. The y axes for endocan in (A) and (B), and the x axis for PCT in (B) are in log values.

Endocan levels at day 0 were not correlated with CRP at day 0 (*r* ^*2*^ = -0.042; *p* = 0.59), or with PCT at day 0 (*r* ^*2*^ = 0.119; *p* = 0.16) (Fig 2). In the other periods, endocan levels were correlated with neither CRP nor PCT (data not shown). These results indicate that the reduction rate in endocan levels during infection was smaller than that of CRP and PCT, and endocan was a biomarker that was independent of the CRP and PCT levels during infection.

### Association of biomarkers with blood culture positivity

**Fig 3:**
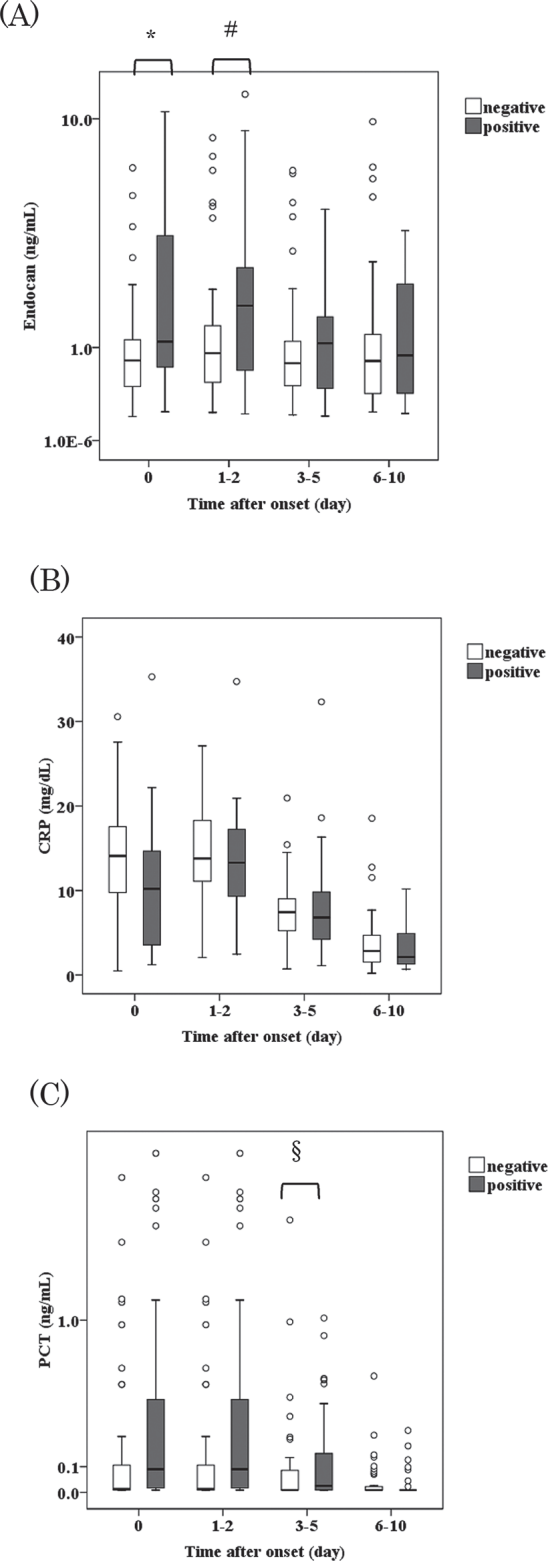
Blood culture positivity and biomarkers during infection. **The boxes represent the interquartile ranges, and the transverse lines in the boxes represent the medians of each group.** The vertical lines represent 1.5 of the interquartile range. The circles indicate outliers of each group. The y axes for endocan in (A) and for PCT in (C), but not for CRP in (B) are in log values.* P = 0.003, # P = 0.02, § P = 0.03

**Fig 4:**
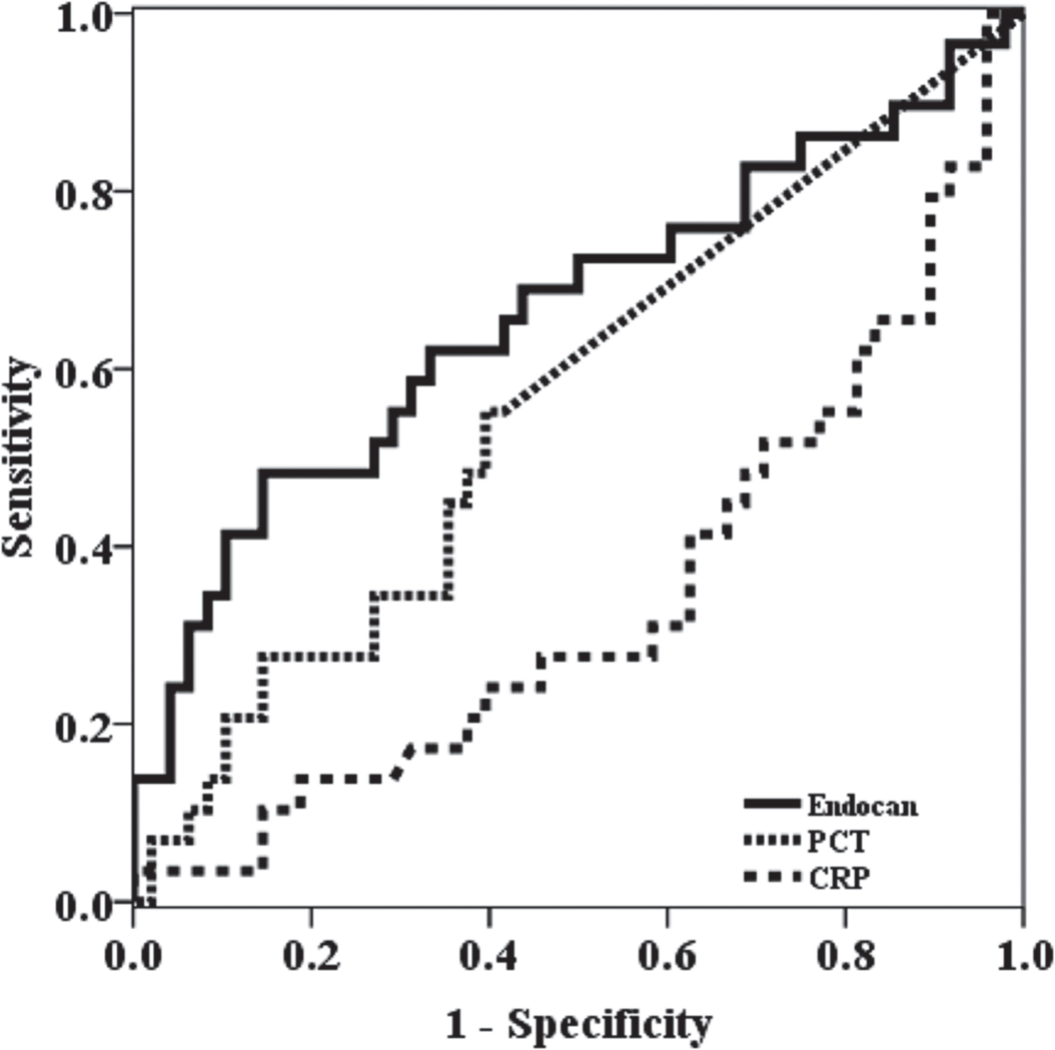
Receiver operating characteristic (ROC) curves for predicting the prognosis of blood culture positivity based on biomarkers levels at day 0. Abbreviations: CRP, C-reactive protein; PCT, procalcitonin

Blood cultures were examined in all 78 patients. Blood cultures were positive in 29 cases and negative in 49 cases. Changes in endocan, CRP, and PCT levels in the positive blood culture groups and negative blood culture groups are shown in Fig 3. Endocan levels at day 0 and at day 1–2 were significantly higher in the positive blood culture group than in the negative culture group (median 1.09 ng/mL vs 0.82 ng/mL, p = 0.003; 1.73 ng/mL vs 0.92 ng/mL, p = 0.02, respectively). CRP did not differ between the two groups during infection. PCT levels at day 3–5 were significantly higher in the positive blood culture group than in the negative culture group (median 0.02 ng/mL vs 0.00 ng/mL, p = 0.03), although PCT levels in other periods were not significantly different between the two groups. Using biomarkers at day 0, receiver operator characteristic (ROC) analysis showed that areas under the ROC curve (AUC) of endocan, CRP, and PCT at day 0 were 0.662, 0343, and 0.563, respectively (P = 0.02, 0.02, 0.35, respectively) (Fig 4). The serum endocan level at day 0 had a specificity of 85.7%, a sensitivity of 41.3%, positive predictive value of 63.2%, and negative predictive value of 76.3% at the cutoff level of 1.70 ng/mL. On the other hand, the serum PCT level at day 0 had a specificity of 83.7%, a sensitivity of 27.6%, positive predictive value of 50.0%, and negative predictive value of 66.1% at the cutoff level of 0.12 pg/mL. Using PCT and endocan, the sensitivity to blood culture positivity was 55.2%.

### Comparisons of clinical factors between patients with high endocan levels and patients with low endocan levels at the onset of infection

According to their serum endocan levels at day 0 with the cutoff level of 1.70 ng/mL, all patients were divided into two groups. As shown in Table 2, there were no significant differences in age, sex, and comorbidities. However, with respect to the primary infection site, the rate of catheter-related blood stream infections was higher in the high endocan group than in the low endocan group (21% vs 3%, P = 0.03). The rate of positive blood cultures was also higher in the high endocan group than in the low endocan group (63% vs 29%, P = 0.01). However, there were no significant differences on multivariate logistic regression analyses, although positive blood culture tended to be related to high endocan levels (odds ratio: 4.24, 95% CI: 0.99–10.34, P = 0.05) (Table 3).

**Table 2:** Clinical characteristics of patients with high endocan levels and patients with low endocan levels at the onset of infection.

Factors	Serum endocan level at onset		P
	< 1.70 ng/mL (n = 59)	≥ 1.70 ng/mL (n = 19)	
Age (years, median, IQR)	79 (67–84)	82 (75–86)	0.14
Sex (male/female)	30/29	10/9	1.00
Comorbidities	-	-	-
Diabetes	17 (28)	3 (16)	0.37
Chronic heart failure	12 (20)	4 (21)	1.00
Cerebrovascular disease	8 (14)	2 (11)	1.00
Chronic obstructive pulmonary disease	6 (10)	2 (11)	1.00
Chronic kidney disease	3 (5)	3 (16)	0.15
Primary infection site	-	-	-
Respiratory tract	29 (49)	6 (32)	0.20
Urinary tract	13 (22)	3 (16)	0.75
Catheter-related bloodstream infection	2 (3)	4 (21)	0.03
Skin and soft tissue	4 (7)	0 (0)	0.57
Intra-abdominal	3 (5)	1 (5)	1.00
Endocarditis	2 (3)	1 (5)	1.00
Meningitis	1 (2)	1 (5)	0.43
Unidentified focus	5 (8)	3 (16)	0.40
Pathogen (s)	-	-	-
Bacteria	31 (53)	12 (63)	0.42
Gram-positive	13 (22)	8 (42)	0.14
Gram-negative	18 (31)	4 (21)	0.56
Fungus	0 (0)	1 (5)	0.24
Polymicrobial	1 (2)	2 (11)	0.15
Unidentified pathogen	27 (68)	4 (21)	0.07
Positive blood culture	17 (29)	12 (63)	0.01
Pitt bacteremia score >4	0 (0)	0 (0)	-

Numbers and percentages of each case are displayed. Abbreviations: IQR, interquartile range.

**Table 3:** Odds ratios for serum endocan level ≥ 1.70 ng/mL in the 78 infected patients.

Factor	Univariate		Multivariate	
	OR (95% CI)	P	OR (95% CI)	P
Positive blood culture	4.24 (1.42–12.67)	0.01	4.24 (0.99–10.34)	0.05
Catheter-related bloodstream infection	7.60 (1.27–45.52)	0.03	3.74 (0.56–25.00)	0.17

Abbreviations: OR, odds ratio; CI, confidence interval.

## Discussion

Widely used infection biomarkers, such as CRP or PCT, have been investigated for their usefulness for distinguishing sepsis from other inflammatory diseases, assessing severity of illness, monitoring effectiveness of treatment, and predicting outcome in many previous studies. Endocan, a newly recognized biomarker, has been investigated only with respect to its relationship with sepsis. Changes in endocan levels during infection and the associations of endocan levels with clinical factors have not been previously investigated.

The first characteristic of endocan identified in the present study was that changes in serum levels during infection were smaller than those of CRP or PCT. PCT and CRP have been recognized as biomarkers with wide ranges in the short course of infection. There is increasing evidence that a PCT-based algorithm is a useful tool to reduce antibiotic duration [16]. The reduction in endocan levels during infection in this study was 22.5%. The serum endocan level has been shown to be related to progression of various cancers, although serum endocan levels were continuously high in these chronic diseases [7,17,18]. The serum endocan level has also been shown to be related to acute diseases other than sepsis, including acute lung injury and acute respiratory distress [19,20], but changes in its serum levels in the acute phase of these diseases have not been investigated. In an *in vitro* study, endocan was continuously produced in the presence of TNF-α in primary cultured HUVECs from 6 to 72 hours, but production over a longer period was not investigated [21]. The process of regulating endocan *in vivo* has not been clarified. From the present results of the dynamics of serum endocan levels during infection, we speculated that the serum endocan level might not only reflect the degree of inflammation, but also the healing process of vascular damage in the late phase of infection.

Biomarker levels with a wide range can be more affected by the timing of sampling or measurement errors. In the Survival Sepsis Campaign Guideline, no recommendation was given for the use of these markers to distinguish between severe infection and other acute inflammatory states [4]. Serum endocan levels might be inappropriate to monitor the effectiveness of treatment, but the mortality in the present study was 1.3%. All the patients in the present study had low Pitt Bacteremia Scores, which means that severe septic cases were not included. In order to investigate whether monitoring serum endocan levels would be useful in assessing the clinical course both in severe cases and in mild cases, more cases of various severities would be needed.

The second characteristic of endocan was that its serum level was not correlated with that of CRP or PCT. In the present study, there were few cases in which both serum levels of endocan and CRP were high. In a previous study of sepsis, endocan was not correlated to CRP and PCT [15], which is compatible with the present results. In clinical settings, some cases of infection can be misdiagnosed because of a low CRP level. The primary production site of CRP is the liver in response to IL-6 [22], and that of PCT is the liver and leukocytes [23]. Endocan was expressed mainly in the vascular endothelial cells, which is different from PCT or CRP. Several cases of catheter-related bloodstream infection showed high endocan levels in the present study. From these results, when the endocan level is high in patients, even if CRP and PCT levels are not high at the onset of the infection, the presence of bacteremia might be predicted.

The third characteristic of endocan was that its serum level was related to bacteremia. For the prediction of bacteremia, PCT has been the most promising parameter. PCT had negative predictive values of 99% and 96% when the PCT cut-off values were 0.5 ng/mL and 0.5 ng/mL, respectively [24]. In a prospective cohort study of SIRS patients, ROC analysis revealed an AUC value of 0.675 for PCT (95% CI 0.636–0.714) for differentiating patients with blood stream infections from those without [25]. In the present study, the PCT level at day 0 was not related to bacteremia. In the ROC analysis of the present study, the AUC value of serum endocan at the onset of the infection was higher than that of PCT for differentiating patients with blood stream infections from those without. When using endocan and PCT for predicting positivity of blood culture, the sensitivity was higher than that of both the biomarkers. There has been no study about usefulness of endocan in bacteremia, so the endocan cutoff value of 1.7ng/ml was determined retrospectively in this study. In order to confirm the cutoff value of endocan, a subsequent prospective study would be necessary.

As previously mentioned, the specific production sites of endocan are endothelial cells. The expression of endocan is up-regulated in response to TNF-α or IL1-β in vitro, although the precise mechanism of endocan expression in infection has not been elucidated [14,26]. LPS also induces endocan release in endothelial cells [15,27]. Inflammatory cytokines were released independent of infection sites. From these results, it appears that, in patients with bacteremia, direct stimulation with pathogenic molecules or inflammatory cytokines in endothelial cells might induce high serum endocan levels in the blood.

Patients with bacteremia were occasionally found to have no focal or systemic manifestations of infection [28]. In previous studies, it has been emphasized that serum endocan levels are related to severity in sepsis. The present results suggest that with measurement of endocan levels at the onset of infections other than sepsis it might be possible to predict bacteremia.

In clinical practice, including cancer management, endocan has not been widely used. It took about three hours to measure the serum endocan level by ELISA assay in the present study. By shortening the measurement time and reducing the cost, endocan might become widely used and recognized as superior to PCT as a screening tool for bacteremia.

There were several limitations in this study. This prospective study was conducted for the purpose of investigating the characteristics of endocan during infection, so that the numbers of enrolled cases may be too small for comparisons of primary infection sites or pathogens. The patients in the present study were admitted to the general ward in our hospital, and the severity of illness was mild or moderate in many cases. Since biomarkers levels including endocan before infection were not measured, endocan levels were not determined at the acute period of infection. It would be necessary to investigate the relationship between each biomarker levels, culture results, and clinical manifestations in hospital-acquired infection. Further study would be needed to assess the whole time course of serum endocan levels and the relationship between endocan levels and bacteremia in severe infection cases.

In conclusion, the serum endocan level was elevated at the onset of infection, and it then decreased slightly during infection. The endocan level was not correlated with the CRP level or the PCT level. In bacteremic cases, serum endocan levels in bacteremia tended to be higher than in non-bacteremic cases. Although endocan level was not identified as a prognostic factor of bacteremia, further prospective study concerning the relationship between serum endocan level and bacteremia would be needed.
